# A faceted approach to reachability analysis of graph modelled collections

**DOI:** 10.1007/s13735-017-0145-8

**Published:** 2017-12-16

**Authors:** Serwah Sabetghadam, Mihai Lupu, Ralf Bierig, Andreas Rauber

**Affiliations:** 1 Institute of Software Technology and Interactive Systems, Vienna University of Technology, Vienna, Austria; 2 Department of Computer Science, Maynooth University, Maynooth, Ireland

**Keywords:** Information Retrieval, Multimodal, Facet, Graph, Recall, Reachability, H.3.3

## Abstract

Nowadays, there is a proliferation of available information sources from different modalities—text, images, audio, video and more. Information objects are not isolated anymore. They are frequently connected via metadata, semantic links, etc. This leads to various challenges in graph-based information retrieval. This paper is concerned with the reachability analysis of multimodal graph modelled collections. We use our framework to leverage the combination of features of different modalities through our formulation of faceted search. This study highlights the effect of different facets and link types in improving reachability of relevant information objects. The experiments are performed on the Image CLEF 2011 Wikipedia collection with about 400,000 documents and images. The results demonstrate that the combination of different facets is conductive to obtain higher reachability. We obtain 373% recall gain for very hard topics by using our graph model of the collection. Further, by adding semantic links to the collection, we gain a 10% increase in the overall recall.

## Introduction

The past decade has witnessed an enormous growth in multimodal digital content, such as video and audio published via social networks. Such information is often interactively annotated and highly connected with related content and their users. This trend sparks the need for a new seamless form of information retrieval that incorporates different modalities for search and other types of information access [1, 29]. Multimodal information retrieval (MMIR) is about extending the classic approach of isolated text, picture and video search into a much more integrated form. Users can express their information need more naturally if they have an option to search with both unimodal and multimodal queries. Likewise, information retrieval systems need to extend search to other modalities to deliver a richer and more diversified range of results, regardless where the search query originated from.

A user who, for example, wants to find songs, videos and reports about *Elvis Presley’s role in Rock music* should be able to express this information need not only in the typical unimodal fashion, that is by keyword search alone or by uploading query song examples. Multimodal search extends the range of options for users, and the same search could then be expressed with the keywords plus a list of pictures on selected albums. One could imagine this being further extended to video clips. Overall, this leads to more flexible forms of expressing users’ information needs.

This area of research presents a great challenge since it deals with several data types and modalities, each with its own intrinsic characteristics and retrieval models. The past decade has witnessed an increased interest in this area of enquiry ranging from associating images with text search scores to the sophisticated fusion of multiple modalities [4, 13, 15, 27, 28, 45].

**Fig. 1:**
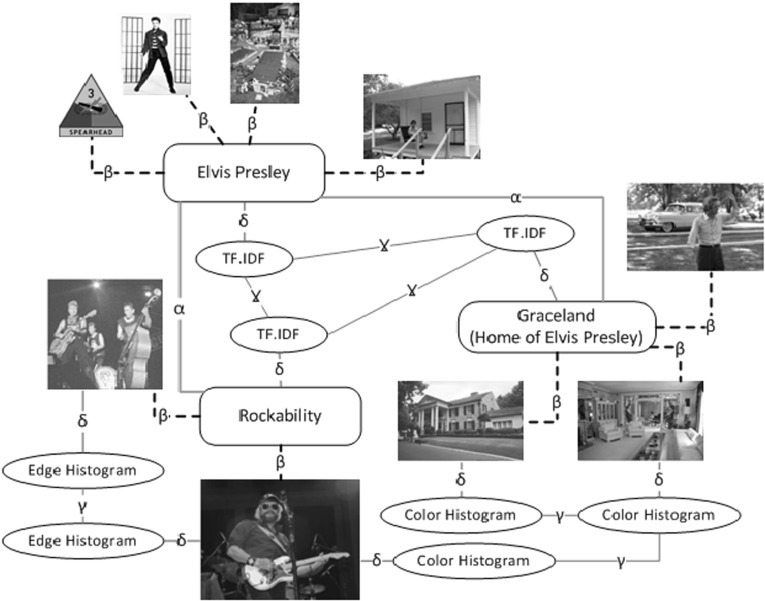
Different types of relations in the model

We differentiate between structured and non-structured MMIR. We call related work which considers only similarity between query and independent information objects as *non-structured MMIR*. However, user-generated multimodal content, domain-specific multimodal collections or platforms like the semantic web impose a new form of *structured MMIR* based on links between different information objects. This is usually represented as a graph of information objects. For our Elvis example, this may look like Fig. 1.

Research in this area mostly focuses on creating a graph of one modality (images or videos) with similarity links between them [18, 48, 49]. However, the data today are multimodal. For instance, shared information about a topic in a social network is frequently a combination of text, images, videos or audio data. To address this challenge, we proposed a graph-based model, named Astera [36, 38]. Previously, we showed that our model can compare favourably with the efficiency of a non-graph-based index, while having the potential to exploit different facets for an improved retrieval performance [35]. In this article, we address the important issue of reaching relevant information objects in the graph. We define this as reachability analysis in the context of this paper. Specifically, we want to explore the following questions:

- How much can we improve reachability of relevant information objects by leveraging multi-facet/poly-representation of query and information objects?
- What is the effect of different facet combinations on recall behaviour of different query categories by their difficulty rating in benchmark evaluations?
- Do different facets with the same recall value visit the same parts of the graph or the same relevant information objects?
- What is the effect of adding semantic and similarity links between information objects?

This article therefore extends our previous work on leveraging multiple facets to improve reachability [37] with a deeper analysis of the proposed model and retrieval methodology in Sects. 3 and 4. We explore a broad set of textual and visual facets and provide an extensive set of experiments on the collection as a complementary set of experiments on reachability analysis that we started before [37]. Previously, we showed the effect of semantic links in reachability. In this work, we consider the effect of similarity links on reachability as well. We compare the contribution of adding these links with the result of adding random links. In addition, a new set of experiments is designed to investigate whether facets with similar recall behaviour visit the same relevant nodes as well. The experiment design and results are described in Sects. 5 and 6. We show the applicability of our model on a multimodal domain by using the ImageCLEF 2011 Wikipedia collection dataset [47]. Finally, our study is summarized in Sect. 7 with an outlook to future research.

## Related work

We divide the related work on MMIR into the two categories of non-structured and structured IR. Traditional IR is based on retrieval from independent documents. We refer to this type of IR as non-structured IR, in which an explicit relation between the information objects is not considered. We provide a brief overview on this part of related work in Sect. 2.1. Leveraging a graph of relations between information objects imposes structured MMIR. We provide the related work in this area in Sect. 2.2.

### Non-structured MMIR

Most popular search engines like Google, Yahoo and Bing build upon text search techniques by using e.g. user-provided tags or related text for images or videos. These approaches have limited access to the data as they completely ignore the information of visual content and the indexes do not contain multimodal information [7, 12, 17, 18, 24]. Another reason is that the surrounding text is usually noisy and this decreases the performance of text-based multimodal search engines. Content-based image retrieval (CBIR) is one of the earliest methods that started to consider image content in the retrieval process. Many systems considered similarity measures only based on the content of the images, which are called pure CBIR systems [6, 26, 33, 42]. One of the earliest research in this category is to utilize low-level feature representation of image content and calculate the distance to query examples to capture similarity. Top documents in the result list are the ones which are visually similar to the query example. On the other hand, systems that include a combination of text and image content in addition to a flexible query interface are considered as composite CBIR systems [16, 21], which comply with the definition of MMIR. This category is suitable for web image retrieval as most images are surrounded by tags, hyperlinks and other relevant metadata. In a search engine, for example, the text result can be reranked regarding the similarity of the results to a given image example of the query. Lazaridis et al. [23] leverage different modalities, such as audio and image, to perform a multimodal search. Their I-Search project is a multimodal search engine, where multimodality relations are defined between different modalities of an information object, for example, between an image of a dog, its barking sound and its 3D representation. They define a neighbourhood relation between two multimodal objects which are similar in at least one of their modalities. In I-Search, however, neither semantic relations between information objects (e.g. a dog and a cat object) are considered, nor the importance of these relations in answering a user’s query.

In addition to using features from different modalities, the interaction process with users is considered in multimodal retrieval as well. Cheng et al. [8] suggest two interactive retrieval procedures for image retrieval. The first method incorporates a relevance feedback mechanism based on textual information, while the second approach combines textual and image information to help users find a target image. Hwang and Grauman [19] have also explored ranking object importance in static images, learning what people mention first from human-annotated tags.

Sometimes in the literature, MMIR is simply called reranking. For instance, Mei et al. [29] definition of reranking (leveraging different modalities, like image or video, rather than only text to find a better ranking) is compatible to what we define as MMIR. They thoroughly survey reranking methods in MMIR and categorize related work in four groups: (1) Self-reranking: mining knowledge from the initial ranked list. (2) Example-based reranking: leveraging few query examples that the user provides along with the textual query. (3) Crowd-reranking: utilizing knowledge obtained from crowd as user-labelled data to perform meta-search in a supervised manner. (4) Interactive reranking which reranks involving user interactions. Based on this categorization, graph-based methods belong to the self-reranking category. Mostly the methods in this category are inspired by PageRank techniques. They create a graph from top-ranked results. Structured IR is similar to this graph-based method, with the difference that the nodes in the graph are not necessarily from top-ranked list.

### Structured MMIR

By structured IR, we denote those approaches that consider the explicit relations between information objects. Usually in such models, a graph is created, which may be based on similarity or semantic relations between information objects. Nodes and edges may hold different definitions in each model. For example, in Liu et al. [25] graph-based model, the video search reranking problem is formulated in a PageRank fashion using random walks. The video sequences are the nodes, and the multimodal (textual and visual) similarity is the hyperlinks. The relevance score is propagated per topic through these hyperlinks.

Jing and Baluja [20] cast the image-ranking problem into the task of identifying “authority” nodes on a visual similarity graph and propose VisualRank to analyse the visual link structure among images. Schinas et al. [40] present a framework to provide summaries of social posts related to an event. They leverage different modalities (such as posts and pictures of the event) to maximize the relevancy of posts to a topic. Clements et al. [9] propose the use of a personalized random walk on a tripartite graph, which connects users to tags and tags to items. The stationary distribution shows the probability of relevance of the items.

Targeting RDF data, Elbassuoni and Blanco [14] select subgraphs to match the query and rank with statistical language models. As a desktop search engine, Beagle++ utilizes a combination of indexed and structured search [30]. Wang et al. [48] propose a graph-based learning approach with multiple features for web image search. They create one graph per feature. Links in each graph are based on the similarity of the images in each graph based on that feature. The weight of different features and relevance scores are learned through this model.

Related research on the ImageCLEF 2011 Wikipedia collection is generally based on a combination of text and image retrieval [47]. To our best knowledge, there is no approach that has modelled the collection as a graph structure and no approach has therefore leveraged the explicit links between information objects and between information objects and their features.

Astera belongs to the structured MMIR category, as it uses a graph of information objects. It differs, however, in the way it creates the graph. Firstly, we include all information objects in the collection, not only the top-ranked results. Top results are used as starting points in traversing the graph. Secondly, different modalities such as image, text, audio and video can be searched in the same graph, whereas related work mostly consider a graph of one modality (e.g. video or image). Finally, we utilize different types of relations between information objects of a collection. In related work, it is only similarity links or only semantic links, whereas we consider both links in the same graph. Further, we add “part-of” and facet links as well. This creates a much richer representation of the complex relationships between information objects.

## Astera information model

We proposed a model to represent information objects from different modalities and their relationships [36, 38]. It is a general framework to compute similarity. We see the information objects as a graph $G = (V,E)$, in which *V* is the set of vertices and *E* is the set of edges. A vertex can represent an information object or its facets. An edge shows different types of relations between two information objects, an information object and its facets, or between two facets.

In this model, we define facet as an inherent feature or property of an information object, otherwise referred to as a representation of the information object. This definition allows considering a document under several points of view, each one being associated with a possible space. For instance, image files which primarily have image facets (comprising several feature spaces such as colour, edge, rotation) have also other facets such as written information (as detected with OCR), time and owner. Each of these is a node linked to the original image object. Each object in this graph may have a number of facets. This has support in the principles of information retrieval, most notably in the theory of poly-representation [22]. The aim is to leverage cognitive and functional representations of information objects to improve IR results. Manifestations of human cognition are different representations of a document, e.g. based on its text or its pictures, whereas a document title, abstract or section headings are considered as different functional representations [41].

We define four types of relations between the objects in the graph [38], forming edges as follows:

- **Semantic** ($\alpha$): any semantic relation between two objects in the collection (e.g. the link between lyrics and a music file). The weight is defined based on the number of semantic relations between two nodes [34]: $w_{ik}=N_{ik}/N_{i}$, where $N_{ik}$ represents the number of information objects that both nodes *i* and *k* are related to, and $N_{i}$ is the number of information objects connected to the node *i*.
- **Part-of** ($\beta$): a specific type of semantic relation, indicating an object as part of another object, e.g. an image in a document. This is a containment relation as an object is part of another one, and therefore we set the default weight to 1.
- **Similarity** ($\gamma$): relation between the facets of the same type of two information objects. The weight is the similarity value between the facets according to some facet-specific metric. For instance, we can compute the similarity between edge histogram facet of two images, or TF.IDF facet of two documents.
- **Facet** ($\delta$): linking an object to its representation(s). Weights are given by perceived information content of features, with respect to the query type. For instance, with a query like “blue flowers”, the colour histogram is a determining facet that should be weighted higher. We find different facet weights experimentally. These weights should be learned for a specific domain, and even for a specific query, e.g. via relevance feedback.

To continue with the user query about *Elvis Presley’s role in Rock music*, we model three related Wikipedia pages (Fig. 1). Here, we show the relations between three documents of *Elvis Presley*, *Graceland* and *Rockability*. The relation between each document and contained images is shown by $\beta$ relations. For example, node *Rockability *(Genre of Elvis’s music) has two images. There is a $\delta$ relation between each document and its TF.IDF facet. The similarity relation between the documents is shown via $\gamma$ relation between their corresponding TF.IDF facets. The similarity relation exists also between the same facets of the images, e.g. between edge histogram of one of *Rockability* images and one of *Graceland* images. These $\gamma$ relations can be between each two images.

In this example, we show only text and image modalities. The example can include video and audio files and their facets as well. Flexibility in defining different nodes and relation types in this model empowers its usage for multimodal retrieval.

Astera model is reported in detail in [35] and [37]. The full implementation of Astera is available online.^1^

## Retrieval methodology

A query may contain different modalities. Our approach is to decompose the query into a list of facets of each modality. For instance, if a query is formed of keywords and image examples, we leverage the combination of its textual and visual facets. However, defining the similarity of a multimodal query with different types of information objects in the graph is not straightforward. We define a function to compute this relevancy. There are two types of vertices in the graph, namely the information objects (which are not directly used for computation) and their facets (which are the actual vertices used for computing similarities). The information object vertices act only as aggregators of weights propagated through the graph via their facet links.

We define the node $v \in V$, where *V* is the set of vertices in the graph. It has facet of $v_{f_i}$, where $v_{f_i} = \{u \in V, \exists \delta (u,v)\}$ and $v = \cup _{i=1}^n v_{f_i}$. For simplicity of notation, we denote the *i*th facet of node *v* as $v_{f_i}$—although it is still a node—and *n* is the number of facets of the node *v*. We define the same for the query *q* as $q = \cup _{j=1}^m q_{f_j}$, where *m* is the number of facets of the query. We define *l* as the number of the common facet types of $\overline{q_{f_i}}$ and $\overline{v_{f_i}}$. We define the function of relevance score value (RSV) as follows:

1 $$\begin{aligned} \hbox {RSV}(q,v) = \sum _{i=1}^l \hbox {norm}(\hbox {sim}(q_{f_i}, v_{f_i})) \cdot w_{f_i}, \end{aligned}$$

where sim is the similarity function between the two facets, norm is the normalizing function, and $w_{f_i}$ is the weight of facet $f_i$ for this query. For similarity function, any standard distance computation can be used. We compute the similarity (sim) between facet values of $q_{f_i}$ and $v_{f_i}$. Usually, the value of a facet is in the form of a feature vector. In case of no common facet, the RSV function output is zero. For normalization, we use the logistic regression formula by Nottelman and Fuhr [31]. It is one of the well-known methods to map the score distribution to probability of relevancy.

For instance, suppose that we search for *Elvis Presley’s role in Rock music* in a collection of documents and images. Each image has a *part-of* relation to its parent document. In addition to the keywords in the query, we have an image example of an album of Elvis Presley. The facets we search are based on are BM25 (as textual facet of the query keywords) and edge histogram (as visual facet of the query image). We find the top 10 documents based on the query keywords and top 10 images based on the similarity of the query image and the images in the collection. Each of these top results receives its corresponding relevancy score based on the normalized similarity score of its facets (Eq. 1). In case, a node is in the top result list of two facets (e.g. an image is selected based on its edge histogram and also BM25 of its metadata), then a weighted normalized combination of the scores is given as the score of this node (Eq. 1). These nodes are the starting points of the graph search in the collection. The scores are propagated to the neighbours based on different edge weights (e.g. similarity or semantic links).

### Traversal method

One of the challenges in graph modelled data is how to “intelligently” traverse the graph and exploit the associations between the information objects. Two well-known methods are spreading activation and Markov chain random walks. Crestani [11] explains spreading activation as a method of associative retrieval to identify relevant information based on the associations between the information elements. Random walks are frequently used in the literature to find relevant information objects in stationary distribution [3, 10, 44]. We found that these two methods are identical [39] if spreading activation complies with the stochastic property in its weight definition. We use spreading activation in our experiments. One reason is our customized definition of weighting on different relation types, which does not necessarily satisfy the stochastic property. Another reason is to apply different constraints such as distance constraint or path constraint in the traversal.

Our hybrid ranking method consists of two steps: First, an initial search with a standard search engine (e.g. Lucene^2^ for text or LIRE^3^) is performed for different facets of a query. We obtain a set of activation nodes from these results. Second, using the initial result set of information objects (with normalized scores) as seeds, we activate the graph from *k* starting points per query facets. Parallel multimodal search is conducted based on graph traversal method. We follow the weighted edges from the initiating points for *t* steps. At the end, we compute the ranked result based on the score value that nodes receive via propagation.

### Facet fusion

To calculate the final scores of the nodes, we need to combine the scores obtained from different facets. Two well-known fusion methods in MMIR are early and late fusion. Early fusion is referred to as fusion in the feature space, unimodal features extracted from different modalities, concatenated into a long feature vector. This is opposed to late fusion, where we integrate the individual results of different features of different modalities. Early fusion is conceptually simpler because it addresses the problem of representation for multimodal objects. However, it suffers from unified representation of the media streams and the curse of dimensionality, whereas late fusion is influenced by the quality of ranking of individual features.

Graph-based approaches can be seen as nonlinear fusion of heterogeneous ranked lists. We perform a form of late facet fusion by combining different weighted facet scores and giving one score to the parent information object. In practice, we have no more a *modality*, but a set of facets/features defining that modality. Starting from different facet results, we integrate results of different facet similarities, then propagate the score to their neighbourhood. After a predefined number of steps, we rank the results based on their final score.

## Experiment design

In this section, we describe the data collection and how we model it in Astera with different link types. We use the ImageCLEF 2011 Wikipedia collection, which is based on Wikipedia pages and their associated images. It comes with 50 topics (i.e. query scenarios/information needs). The topics are divided into four categories of easy (17 topics), medium (10 topics), hard (16 topics) and very hard (7 topics) [47]. They cover types of queries that are supposedly better solved by textual, visual or multimodal retrieval. It is a multimodal collection and an appropriate choice for testing the rich and diverse set of relations in our model. The goal of this benchmark setting is to retrieve images. Each image has one metadata file that provides information about name, location, one or more associated parent documents in up to three languages (English, German and French), and textual image annotations (i.e. caption, description and comment). The collection consists of 125,828 documents and 237,434 images. Based on the image metadata, we created nodes for images, parent documents and corresponding facets. We enriched the collection by adding various link types as follows.

*Facet links* The facet ($\delta$) relation between each information object and its corresponding facets are added to the graph. We extract facets from both images and documents. We utilize default configuration of a Lucene indexer for three document textual facets of TF.IDF (term frequency times inverse document frequency as the weighting scheme in the vector space model), BM25 (best match version 25, the main instantiation of probabilistic relevance framework) and LM (language modelling).

For images, we use both visual and textual facets. As for visual facets, we use the four image features provided by the collection. They are CEDD (color and edge directivity descriptor), CIME (a border/interior classification algorithm which classifies pixels into interior or border [43]), SURF (speeded up robust features is a performant scale- and rotation-invariant interest point detector and descriptor) and TLEP (LBP texture descriptor [32], which describes the spatial structure of a local texture) features. For textual facets of the images, we index the metadata XML files of each image. These files include textual annotations (caption, comment and description) of images. Using Lucene, we index them as separate fields and search based on a multi-field indexing. We use TF.IDF, BM25 and LM facets.

*Semantic links* We use Linked Open Data to add semantic links. We map the correspondent version of DBpedia for Wikipedia 2011. We observed from DBpedia that there are representations of different concepts in one language, as well as representations of the same concepts in different languages. We call the links between the concepts in one language intralingual semantic links and between the same concepts in different languages interlingual semantic links.

The collection documents are in three languages, and we use only English queries in current experiments. Therefore, we consider interlingual semantic links to connect documents from different languages. For choosing semantic links, we consider the statistics Tonon and colleagues [46] provided on different link types. They rank all properties by their observed likelihood of leading to relevant entities. Their results demonstrate that links like <DBpedia:wikilink> are too general links that broaden to non-related objects. However, links like *sameAs* are more promising. It leads to better precision as they refer to the same or similar real-world entity [46].

To add sameAs link, we use the interlingual link information from DBpedia links. This information is not available for the working dump version 3.4. We use version 3.8 which includes this information. For each English document, we found the correspondent triple in French and German languages. This triple is added via sameAs link to the collection. We added 29,828 available links for EN-FR documents, 34,530 links for EN-DE documents and 36,295 between FR and DE documents.

*Similarity links* Similarity links ($\gamma$) connect the same facet type of two information objects. Theoretically, we can add a similarity link based on a specific facet between an information object and all other information objects in the collection. These connections create a highly connected graph. However, there would be a lot of weak similarity links in such a graph. To filter weak links, we set a limitation for adding a similarity link. For current experiments, we take top 10 similar neighbours of documents/images based on their textual/visual facets. The weight of these links is the similarity between an information object and its neighbour.

We perform similarity computations separately for English (EN), German (DE) and French (FR) annotations as textual facets of each image. For example, we get the French comment, caption and description of an image, create a document of these fields, and find similar images based on this document. An image may have 30 similar neighbours in the case of having annotations in all languages. We perform the same scenario separately for documents, retrieving top 10 similar ones in each language. In total, we added 3,535,437 similarity links in the graph.

## Experiment results

We start the experiments by exploring the distribution of relevant objects in the graph (Sect. 6.1). We consider the effect of poly-representation in the facet dimension in reachability in Sect. 6.2. Continuing the experiments in Sects. 6.3.1 and 6.3.2, we consider the reachability by including semantic and similarity links.

### Relevant objects distribution

As a first experiment, we want to obtain an understanding of the collection in particular of how the relevant images are distributed in the graph. The relation types used in this section are part-of and facet links, which are the basic connections needed to model the collection. There are 50 topics in ImageCLEF 2011 Wikipedia collection. We perform the traversal up to 40 steps for each of these topics, as no more new nodes are visited after this step in the graph. In each step, we check if we visit new relevant images for a specific topic.

**Fig. 2:**
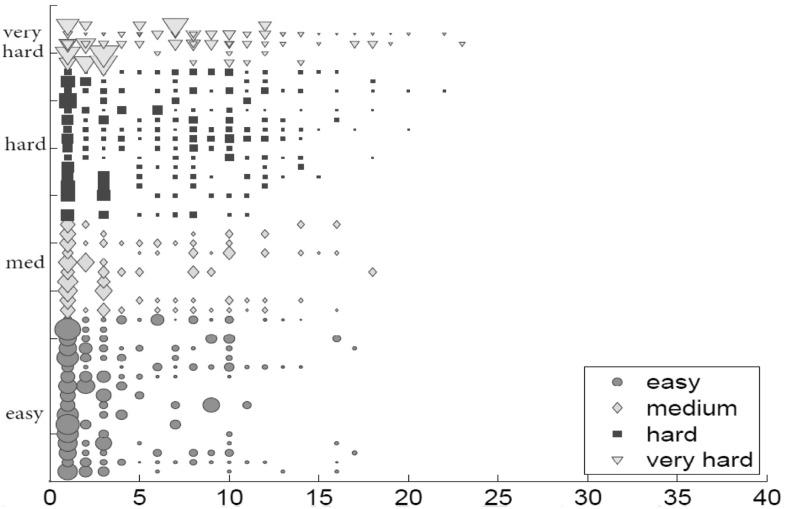
Overall recall aggregated per category over steps

Figure 2 shows the distribution of relevant nodes in the collection as we start from three facets, document textual facet ($\hbox {TF}.\hbox {IDF}_\mathrm{D}$), image visual facet (CEDD) and image metadata textual facet ($\hbox {LM}_\mathrm{I}$) —as a sample of each category of facets. The x axis is the number of steps we traverse the graph, and y axis is the ID of the query topics. In each step, we count the number of new relevant images we visit. Existence of a shape (circle/square/star/triangle) indicates visiting at least a true positive. The size of a shape indicates the fraction of new relevant images reached in the respective steps, calculated as:

2 $$\begin{aligned} \hbox {size} = \frac{\text {number of relevant nodes seen in this step}}{\text {total number of relevant nodes}} \end{aligned}$$

for a query topic ID.

We observe a large number of large shapes in the first steps. This observation shows that we visit more relevant images as we start the traversal initiated from different facet results. We observe that relevant images for easy and medium topics (circles and diamonds) are mostly reachable at the very beginning steps, whereas for hard and very hard topics (squares and triangles) they are more reached in later steps during graph traversal. They show almost constant increase as we traverse the graph.

### Reachability from different facets

In this section, first we investigate the effect of individual and multiple facets in improving recall. Then, we consider which parts of the graph they visit. Finally, we investigate the effect of different facets and graph traversal on recall of different topic categories. These experiments are based on the graph model of the collection including only part-of and facet relations, but not semantic or similarity links, which we investigate in Sect. 6.3

#### Different facet combination

We investigate the recall behaviour of textual and visual facets and a variety of combinations. For each facet (e.g. $\hbox {TF}.\hbox {IDF}_\mathrm{D}$), we use a standard search engine to find the top 10 results. The reason to choose top 10 is the usual way a user looks at popular search engine results. These results are used as starting points for traversing the graph. We calculate recall in each step based on all the nodes visited up to this step.

In our previous work [38], we investigated recall behaviour only for TF.IDF facet. In this paper, we investigate a wider range of facets which are cognitively or functionally different. For visual facets [35, 36], the experiments were only based on the CEDD facet. In this section, we add three other image features provided by the collection: SURF, CIME and TLEP.

**Fig. 3:**
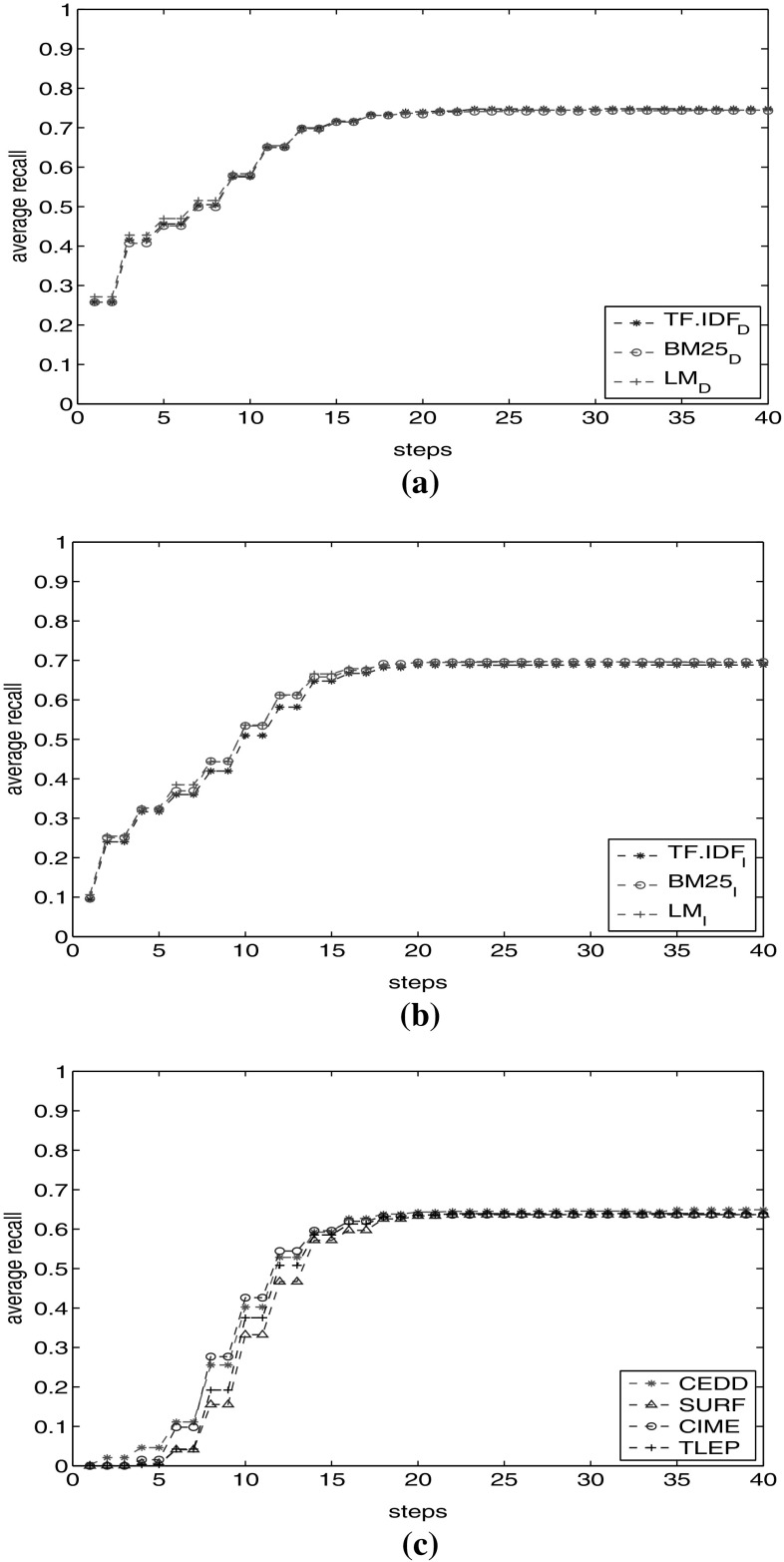
Recall with different document or image facets. **a** Recall with different document textual facets. **b** Recall with different image textual facets. **c** Recall with different image visual facets

First, we show results of all three textual facets (TF.IDF, BM25 and LM) for both documents and images. Figure 3a shows the recall for document textual facets. We observe similar behaviour of different document textual facets, with a high shift in recall in the starting steps. Next, we perform the same analysis for image textual facets (Fig. 3b). We observe that they show the same recall behaviour as well, starting from lower values. However, $\hbox {LM}_\mathrm{I}$ and BM25$_\mathrm{I}$ show slightly higher recall than $\hbox {TF}.\hbox {IDF}_\mathrm{I}$. Finally, we show individual effects of visual facets on recall in Fig. 3c. All four image facets demonstrate approximately the same behaviour. Except CEDD, the facets do not visit relevant images in the first five steps.

According to the analysis so far, each category of facets (document textual, image textual and image visual facets) shows approximately the same recall behaviour. For further investigations on the effect of facet combinations, we select a representative from each category for a more detailed analysis in upcoming experiments. The reason is to limit the starting points to investigate the effect of the graph structure and links on recall. If we start from many points, we visit a large number of nodes in each step. We thus select only one facet for each category ($\hbox {TF}.\hbox {IDF}_\mathrm{D}$ from document textual facets—as it is frequently used in the IR literature, and $\hbox {LM}_\mathrm{I}$ from image metadata textual facets—as it is based on completely different algorithm compared to TF.IDF to investigate the poly-representation principle in our experiments). Similarly for image visual facets, we select CEDD. According to Fig. 3c, it shows slightly better recall at first steps. Further, CEDD has shown better results compared to other visual facets from ImageCLEF 2011 Wikipedia Collection [2].

**Fig. 4:**
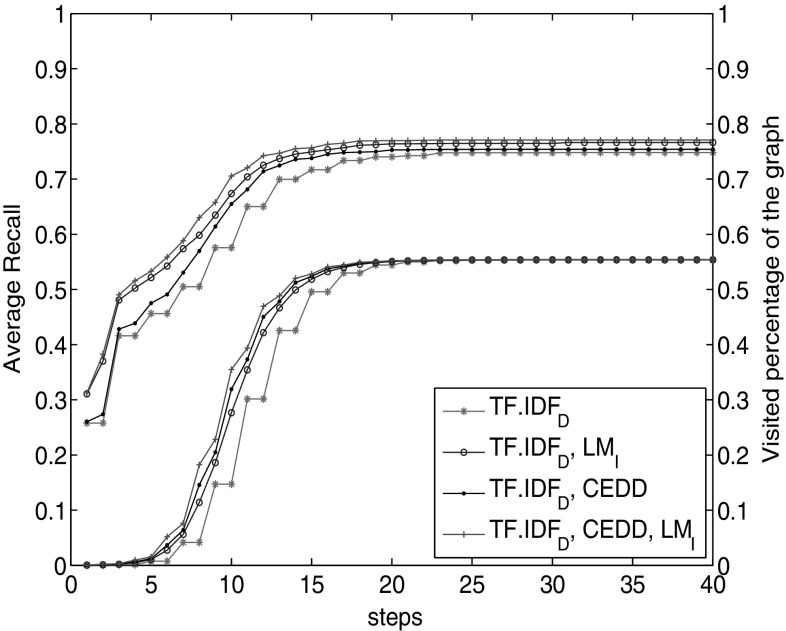
Average recall obtained compared to the percentage of the graph seen through different facet combinations

We perform the experiment for different combinations of these three facets in (Fig. 4). The upper set of lines shows the average recall obtained through each combination. The pink line with stars shows the recall with $\hbox {TF}.\hbox {IDF}_\mathrm{D}$ facet. It is the basis for our comparison. The recall results of $\hbox {TF}.\hbox {IDF}_\mathrm{D}$-CEDD are nearer to those of $\hbox {TF}.\hbox {IDF}_\mathrm{D}$, while $\hbox {TF}.\hbox {IDF}_\mathrm{D}\hbox {-}\hbox {LM}_\mathrm{I}$ obtains higher recall values. It is closer to those obtained with using all facets $\hbox {TF}.\hbox {IDF}_\mathrm{D}\hbox {-}\hbox {LM}_\mathrm{I}$-CEDD.

To clarify whether recall increase is the effect of simply visiting more nodes or it is because of traversing through a meaningful path in the graph, we added another experiment to Fig. 4. The lower set of lines indicates the percentage of the graph visited through each combination of these facets. Looking at this set of lines, we observe that with $\hbox {TF}.\hbox {IDF}_\mathrm{D}$-CEDD combination we visit a higher percentage of the graph compared to $\hbox {TF}.\hbox {IDF}_\mathrm{D}\hbox {-}\hbox {LM}_\mathrm{I}$. This is opposed to their recall lines, where $\hbox {TF}.\hbox {IDF}_\mathrm{D}\hbox {-}\hbox {LM}_\mathrm{I}$ facet outpaces the $\hbox {TF}.\hbox {IDF}_\mathrm{D}$-CEDD combination. This observation confirms that not necessarily visiting more number of nodes results in better recall. It is important to choose facets effectively. Clearly, with $\hbox {TF}.\hbox {IDF}_\mathrm{D}\hbox {-}\hbox {LM}_\mathrm{I}$ combination, we visited fewer nodes but obtained higher recall.

Again in Fig. 4, we observe that the combination of the three facets shows higher recall value than the other two combinations. This highlights the importance of different, diverse representations of the query to reach more relevant objects.

**Fig. 5:**
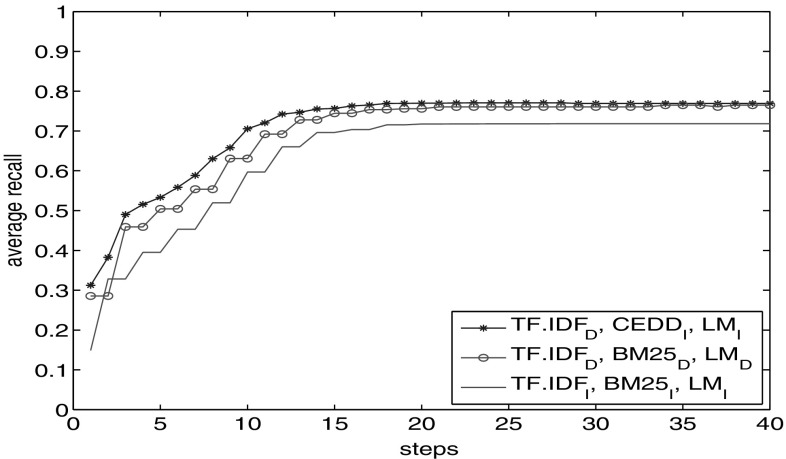
Recall comparison of combination of document textual facets with image textual facets and with $\hbox {TF}.\hbox {IDF}_\mathrm{D}$, CEDD, $\hbox {LM}_\mathrm{I}$ combination

In the next experiment, we compare the result with the diverse facet combination of $\hbox {TF}.\hbox {IDF}_\mathrm{D}$, $\hbox {LM}_\mathrm{I}$ and CEDD (Fig. 5). We observe that recall with document textual facets (the line with circles) outpaces the image textual facets. However, the combination of the three diverse facets outperforms the other two. This again confirms the effect of leveraging diverse sets of facets towards better reachability.

#### Qualitative analysis

In the previous section, we observed very similar recall behaviour of different visual and textual facets (Fig. 3). The question arises whether with the same recall value, we visit the same relevant nodes as well. For this purpose, we calculated the percentage of the part of the graph which is visible only to a specific facet and not to any others. We calculate the percentage of different nodes visited in a step as:

3 $$\begin{aligned} d_{f_i} = \frac{|C_{f_i}|-|M| }{|C_{f_i}|} , \end{aligned}$$

where $C_{f_i}$ represents the nodes seen in a step for facet $f_i$, and $M = \bigcup _{f_j\in F \setminus \{f_i\}} {C_{f_i} \cap C_{f_j}}$, where *F* is the set of facets and *M* is the set of nodes also seen by other facets. This way, by $|C_{f_i}|-|M|$ we count the nodes only reachable through the facet $f_i$. The value $d_{f_i}$ is the ratio of nodes reachable only through this facet. We calculate this ratio for visited *relevant* images as well. In this case, $C_{f_i}$ is all the relevant images seen in a step for facet $f_i$.

**Fig. 6:**
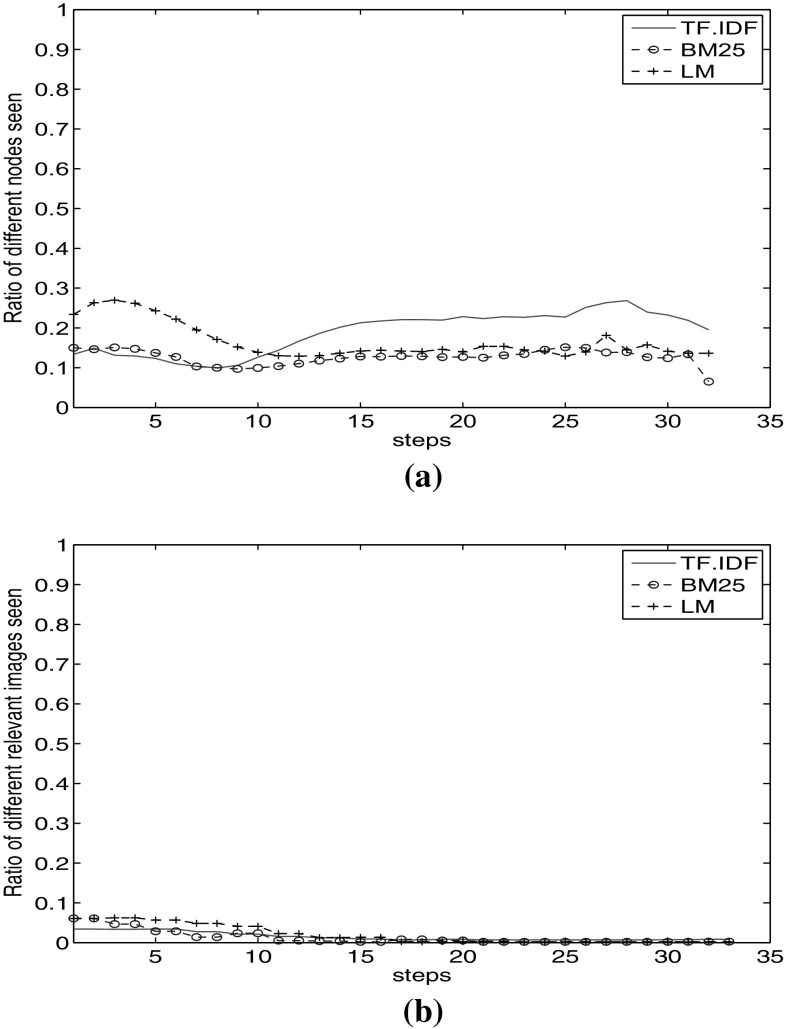
Ratio of different nodes visited from document textual facets. **a** Ratio of different nodes visited through document textual facets. **b** Ratio of different relevant images visited through document textual facets

**Fig. 7:**
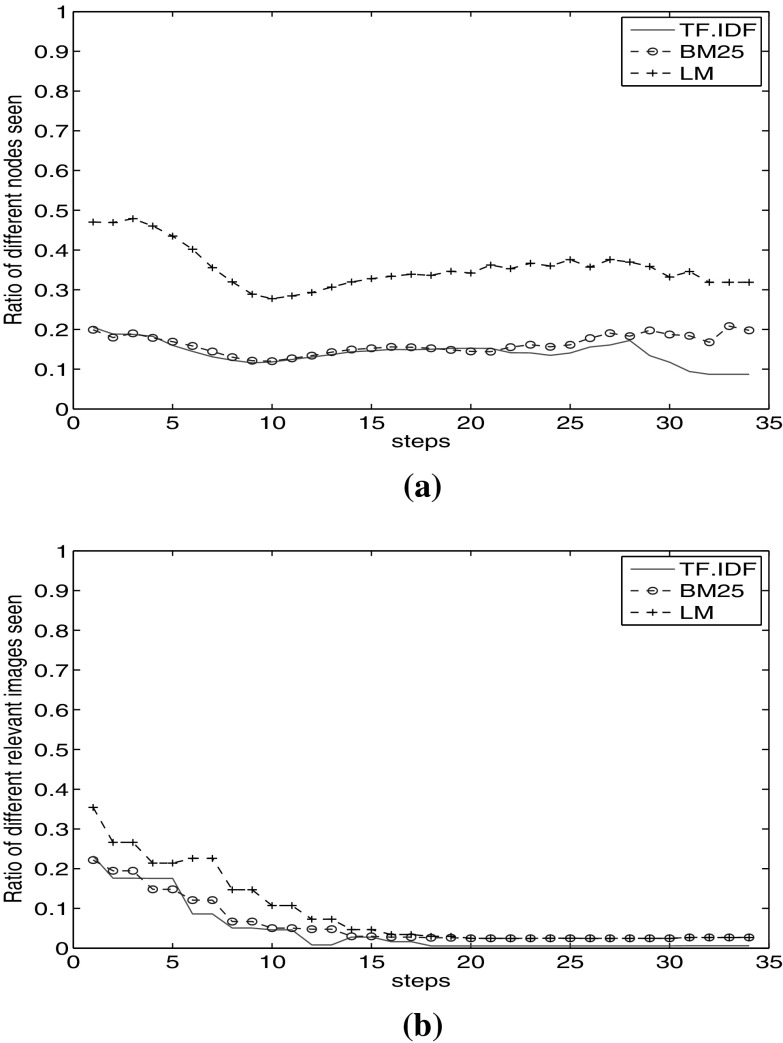
Ratio of different nodes visited from image textual facets. **a** Ratio of different nodes visited through image textual facets. **b** Ratio of different relevant images visited through image textual facets

**Fig. 8:**
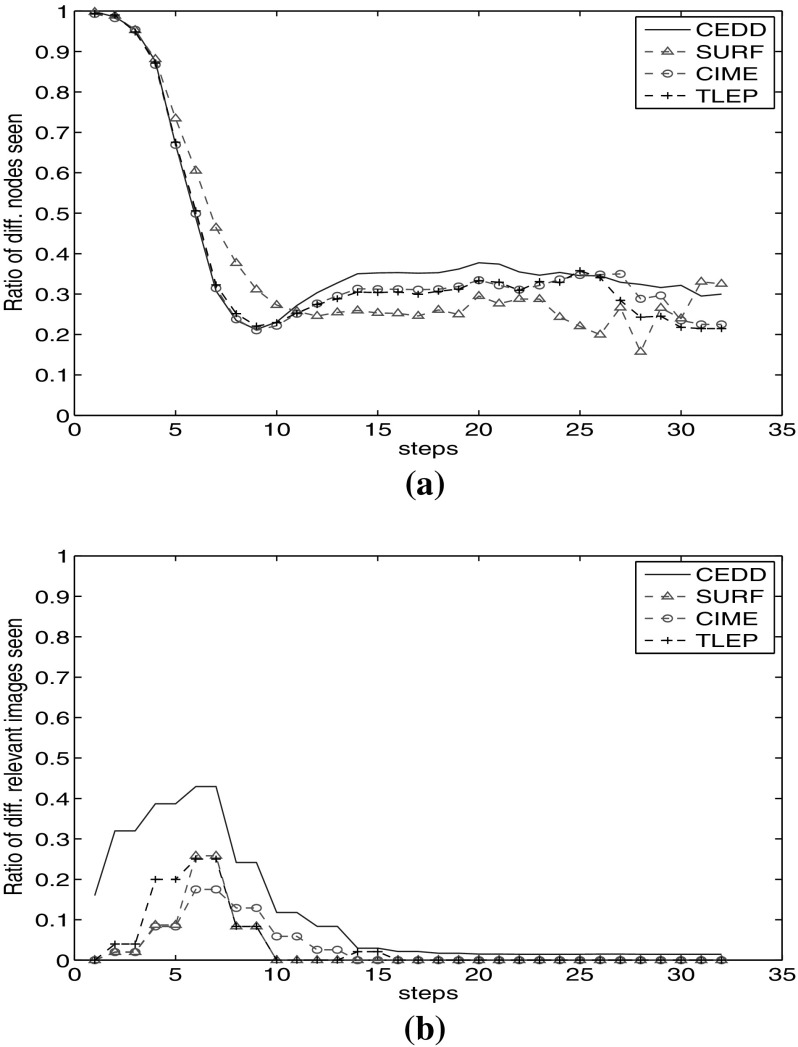
Ratio of different nodes visited from image visual facets. **a** Ratio of different nodes visited through image visual facets. **b** Ratio of different relevant images visited through image visual facets

We start with the nodes visited from the result of document textual facets. We perform this experiment with $\hbox {TF}.\hbox {IDF}_\mathrm{D}$, BM25$_\mathrm{D}$ and $\hbox {LM}_\mathrm{D}$ facets. Figure 6a shows that in the steps 1-10, $\hbox {TF}.\hbox {IDF}_\mathrm{D}$ and BM25$_\mathrm{D}$ show the same behaviour. They visit in average 13% different nodes compared to the other two, while $\hbox {LM}_\mathrm{D}$ starts from different nodes in the graph. After the 10th step, $\hbox {LM}_\mathrm{D}$ and BM25$_\mathrm{D}$ show the same behaviour, where $\hbox {TF}.\hbox {IDF}_\mathrm{D}$ visits 20% different nodes. However, we observe in Fig. 6b that all these three facets visit approximately the same relevant images. They show small differences at the beginning, but after 5 steps, LM keeps visiting different relevant images.

Figure 7a shows the ratio of different visited nodes through $\hbox {TF}.\hbox {IDF}_\mathrm{I}$, BM25$_\mathrm{I}$ and $\hbox {LM}_\mathrm{I}$ facets of image metadata. We observe that $\hbox {TF}.\hbox {IDF}_\mathrm{I}$ and BM25$_\mathrm{I}$ start with 20% different nodes in the graph. Each keep visiting on average 15% different nodes compared to the other two facets. However, $\hbox {LM}_\mathrm{I}$ proposes a rather divergent view. It starts with 48% different nodes. It starts with different top results and keeps this different path to the end. Its impact on visiting relevant images is clear in Fig. 7b. Up to step 15, $\hbox {LM}_\mathrm{I}$ visits more different relevant images than the other two facets.

From the results of document and image textual facets (Figs. 6a, b and  7a, b), we observe that the LM facet shows different behaviour compared to TF.IDF and BM25. A probable reason is the structural difference of these facets. Both TF.IDF and BM25 are based on *tf* and *idf* factors, while LM holds a completely different probabilistic view.

Figure 6a shows a lower ratio of different images visited compared to Fig. 7a. When we start the traversal based on document textual facets (Fig. 6a), we traverse less divergent parts of the graph, compared to the graph parts visited as we start with image textual facets (Fig. 7a). The reason is that starting with document textual facets, we touch documents in the first place. In each next step, we see all images inside a document, covering a large number of images in one step, whereas starting with image textual facet results, we touch individual images in the first step. These images may be part of different documents which lead to the broader view to the graph. This results in visiting more divergent relevant nodes, as we observe large difference in starting steps in Fig. 6b compared to Fig. 7b. We observe that the LM facet not only visits different parts of the graph, but also keeps visiting different relevant images. This observation reinforces the poly-representation principle [22] in using cognitively dissimilar facets. The reason is that BM25 and LM use different approaches for computing the relevancy of a document.

We calculate the $d_{f_i}$ values (Eq. 3) in each step for all four image visual facets (Fig. 8a). We observe that each of these facets starts from different parts of the graph ($d_{f_i} = 1$). However, it decreases with a high slope to $d_{f_i} = 0.2$ after 10 steps. Afterwards, they keep visiting different nodes with different ratio (between 20 and 35%). Facets CIME and TLEP show very similar behaviour, while CEDD and SURF diverge more.

Figure 8b shows the ratio of relevant images seen through each of these facets. In the first steps, each facet visits different relevant nodes, of which CEDD visits higher number of relevant nodes which are not reachable by other facets until step 17. After step 18, relevant nodes seen through CIME, TLEP and SURF show overlap with seen relevant nodes by CEDD. However, CEDD keeps visiting 2% different relevant nodes.

Comparing Figs. 3c and 8a, we observe that although visual facets show the same recall value, they visit different relevant nodes at least in the first 15 steps.

#### Topic categories analysis

From Fig. 2, we observed that we visit more relevant nodes, especially for hard and very hard topics, as we traverse the graph. In this experiment, we show the effect of different facet combination on recall behaviour of the topic categories (easy, medium, hard, very hard [47]).The three representative facets $\hbox {TF}.\hbox {IDF}_\mathrm{D}$, CEDD and $\hbox {LM}_\mathrm{I}$ are used.

**Fig. 9:**
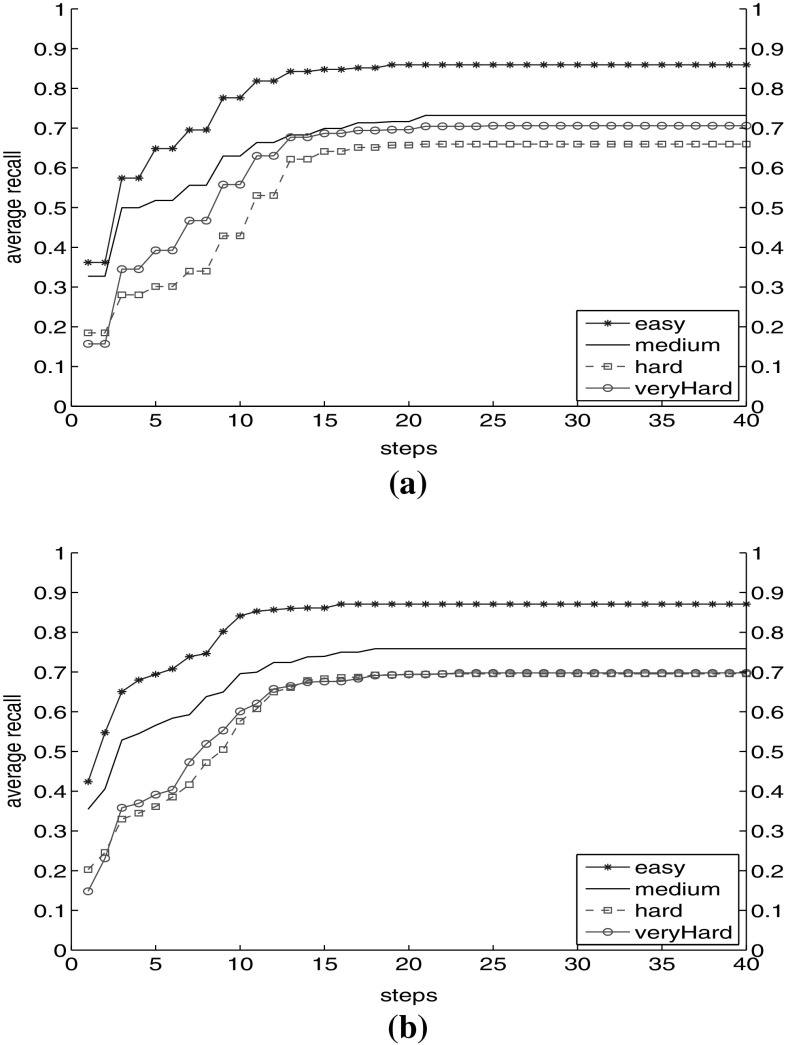
Average Recall in the base graph (with facet and part-of relations) on different categories of topics. **a** Average recall using $\hbox {TF}.\hbox {IDF}_\text {D}$. **b** Average recall using $\hbox {TF}.\hbox {IDF}_\text {D}$, CEDD, $\hbox {LM}_\text {I}$

In the first experiment, we include only $\hbox {TF}.\hbox {IDF}_\mathrm{D}$ results to start the search in the graph. Figure 9a shows the average recall for different categories. One observation is the increase rate in each category. Easy topics show the increase rate of 138% (from 0.36 to 0.86), where it is 128% for medium topics (from 0.32 to 0.73), 266% for hard topics (from 0.18 to 0.66) and 373% for very hard topics (from 0.15 to 0.71). The values show that easy and medium topics are apparently answerable by direct querying, while it is in the hard and very hard topics that the graph model shows most promise. Another observation is that all categories of topics reach a plateau after step 21 (easy topics with 0.86, medium topics with 0.73, hard and very hard topics with 0.66 and 0.71). An interesting observation is the behaviour of very hard topics after the 3rd step: they outpace hard topics. This demonstrates that as we go farther in the graph, we cover a higher percentage of recall for very hard topics rather than hard topics.

Reaching the recall plateau after 21 steps has two messages for us. First is that by conducting the traversal, we can expect increase in recall in the graph to about 21 steps. The reason is that we are still visiting relevant nodes as we go farther in each step. Second is that after the 21st step we do not visit relevant images any more, and recall is still less than 0.73 even for medium topics. This shows that the graph is not connected. Our log files show that no more new nodes are reached after the 40th step for any of the topics. Therefore, the probability of continuing the traversal and seeing new relevant new nodes is very unlikely (when using only the textual facet in this set-up).

As second experiment, we start from the result of all three facets to start the propagation (Fig. 9b). We observe higher recall in steps 1–10. In addition, the recall plateau in each category can be reached earlier with all facets. We have the same values between steps 5 and 10 comparable to the recall value with $\hbox {TF}.\hbox {IDF}_\mathrm{D}$ facet for steps 10–15. We visit higher number of relevant nodes in fewer steps of traversal. Further, recall has increased to 0.71 for hard topics. The final recall for different categories did not show a significant difference. The reason is the connectivity of the graph that after a number of steps, all reachable relevant nodes are visited.

In these experiments, the total number of the nodes seen at plateau is in average 178,620 nodes, which is about half of the collection size. This illustrates that we have access to almost half of the graph. The convergence of traversal performance at about 21st step is another confirmation that we do not have access to all the graph. To tackle this limitation, we continue the experiments by adding semantic and similarity links to the collection to increase reachability of relevant nodes.

### Reachability through different links

Up to here, we used various facets in our recall investigation, leveraging only part-of and facet links. Now, we investigate the role of adding further links to improve reachability. All experiments are started based on the set of top 10 results of standard search on $\hbox {TF}.\hbox {IDF}_\mathrm{D}$, CEDD and $\hbox {LM}_\mathrm{I}$ facets as in previous experiments.

**Fig. 10:**
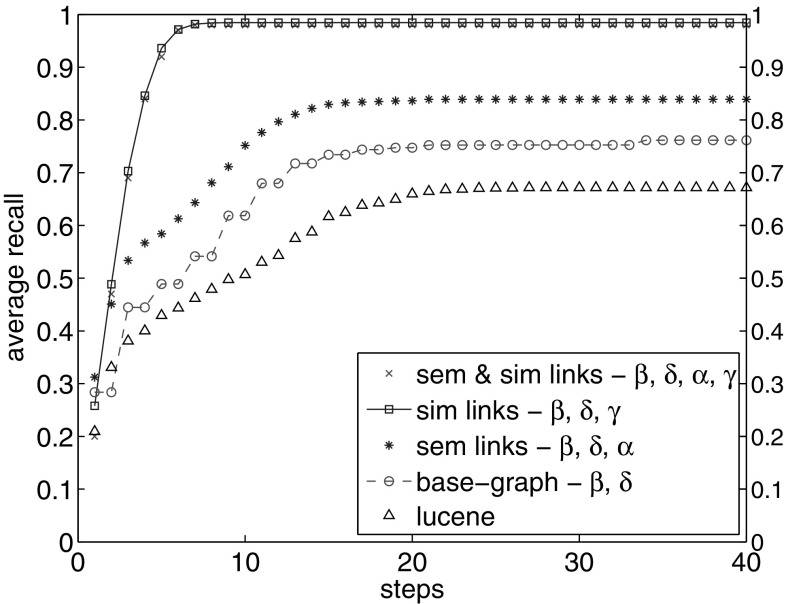
Average recall of all topics after 40 steps with different links

Before adding any links to the collection, we compare the recall of our graph model of the collection with Lucene results. One claim could be that going through Lucene results, we could reach the same recall. The baseline recall is what we had in Fig. 3a with $\hbox {TF}.\hbox {IDF}_\mathrm{D}$ facet. It is shown as *base graph* line in Fig. 10. We observe that this line reaches a plateau after step 17, with 0.76 of recall.

In order to compare with Lucene results, we take the same number of results as the number of new nodes seen in each step in the base graph. For instance, if we visit 430 new nodes in the third step, we add the subsequent 430 results from the ranked list, calculating the recall at each step. Figure 10 shows that *base graph* line outpaces Lucene results after 3 steps. It reaches to the plateau of 0.76 compared to the recall value of Lucene results with plateau of 0.66 (Fig. 10).

#### Semantic links

We add semantic links to the collection based on the DBpedia dump (Sect. 5). We calculate recall in each step (Fig. 10). We observe a steeper slope in the first steps, up to 0.84 recall in the 15th step. We obtain 10% increase in the overall recall. This demonstrates that adding semantic links leads to higher reachability and to a larger number of relevant nodes.

**Fig. 11:**
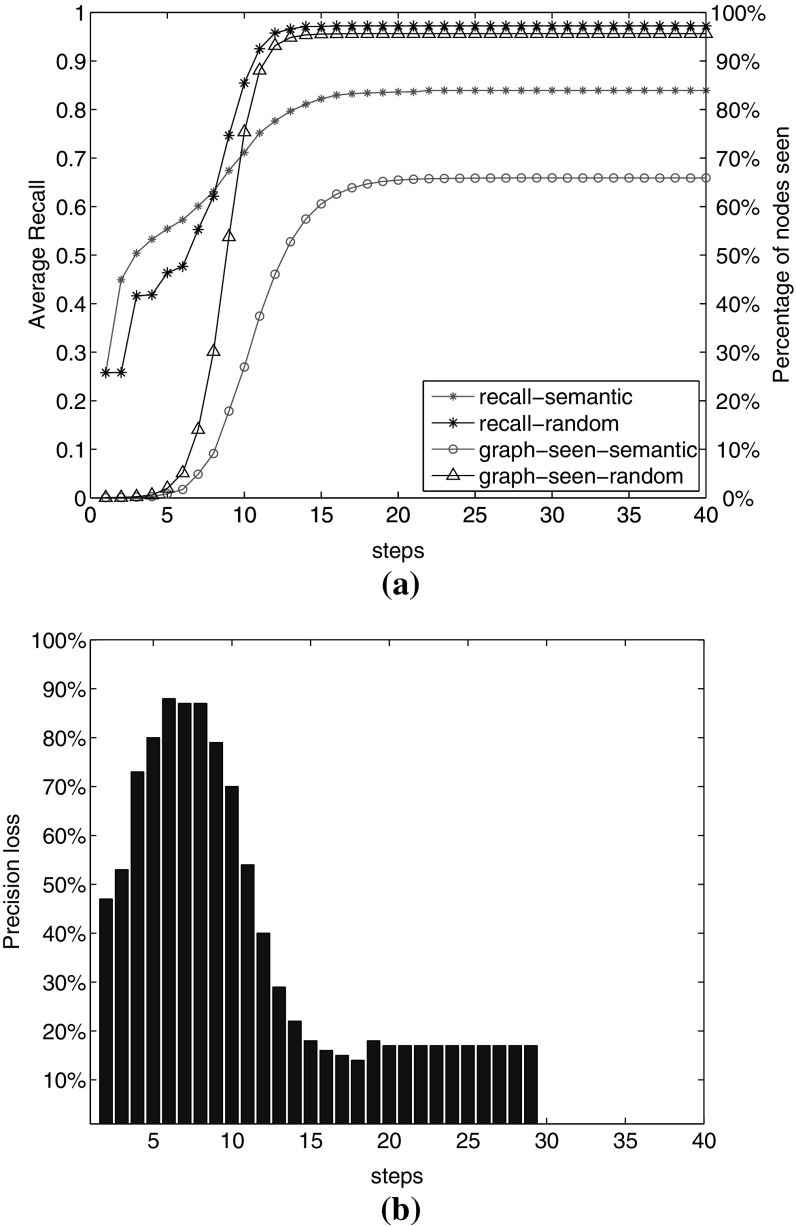
Comparing the effect of adding semantic links and random semantic links on recall and precision loss in the graph. **a** Recall and graph percentage seen from adding semantic links compared to added random links. **b** Precision loss in each step: comparing precision value with random semantic links to the value with real semantic links. For instance, we observe 88% precision loss in the 6th step

We gain 10% increase in recall by visiting 217,395 nodes. It happens by visiting 22% increase in the number of visited nodes. This observation shows that it is not easy to increase recall when the baseline is already 0.76. It has also the overhead of visiting a large number of nodes. However, the question arises whether *any* additional set of links would lead to such an improvement. Do we obtain the same recall value by adding the same number of *random* links? To examine, we compare the result of adding semantic links with adding the same number of random links between EN-DE, EN-FR and DE-FR documents, as we did in Sect. 5. Figure 11a shows this comparison. We observe that after 12 steps with random semantic links, we reach a very high recall of 0.97, by visiting 95% of the graph nodes. However, we reach 0.84 recall with real semantic links by visiting only 66% of the graph. Visiting large number of nodes requires high computational resources, which affect the graph traversal speed.

Further, we compare the amount of precision we lose, with the increase rate of the number of visited nodes by visiting virtually the entire graph depicted in Fig. 11b. We calculate the precision loss as:

4 $$\begin{aligned} \text {precLoss}= 1-\left( \frac{\text {precision with random semantic links}}{\text {precision with real semantic links}}\right) . \end{aligned}$$

We observe precision loss of more than 50% in steps 2–11. For instance, in step 6, we lose up to 88% precision compared to using real semantic links. This step shows that adding semantic links contributes to higher recall with meaningful links. The precision loss from these additional links is lower than if we had added the same number of links randomly.

#### Similarity links 

**Fig. 12:**
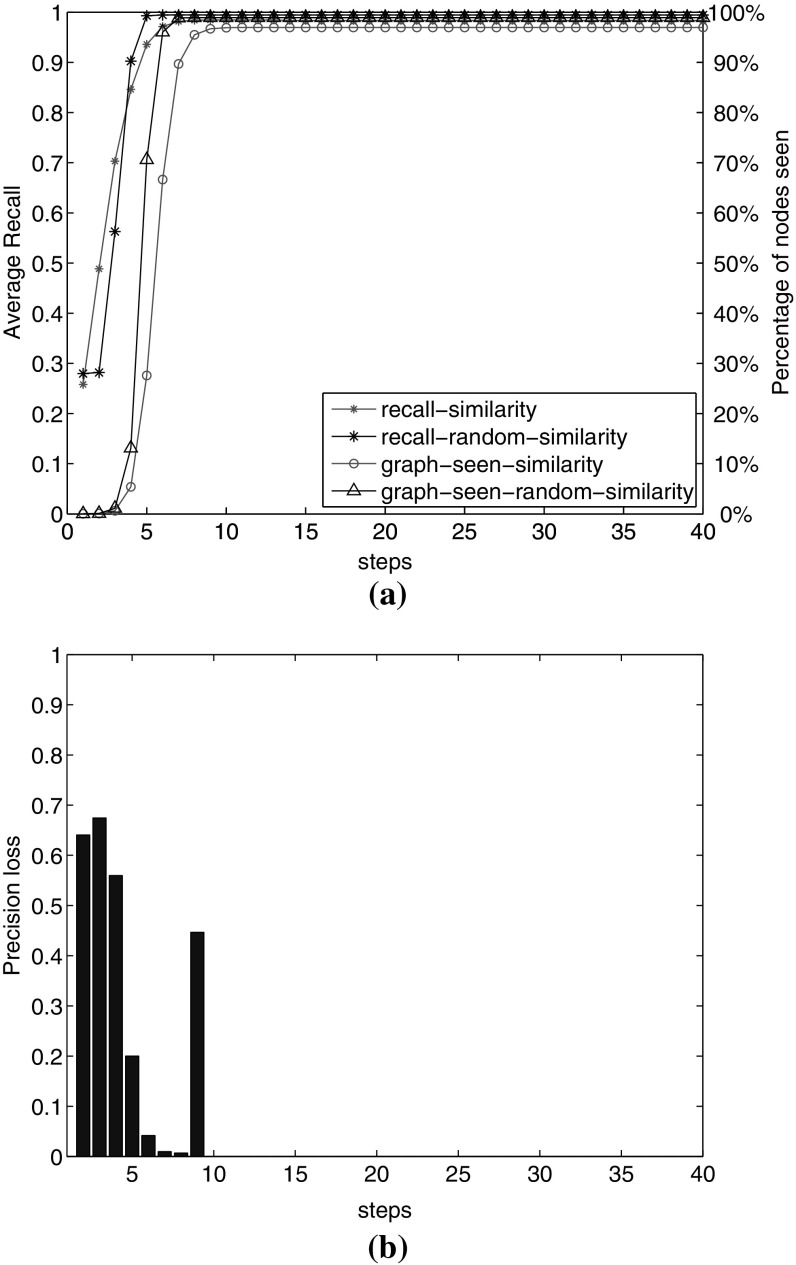
Comparing the effect of adding similarity links and random similarity links on recall and precision loss in the graph. **a** Recall and graph percentage seen from adding similarity links compared to added random links. **b** Precision loss in each step: comparing precision value with random similarity links to the value with real similarity links. For instance, we observe 67% precision loss in the 2nd step

In this experiment, we add the similarity links to the graph as described in Sect. 5. Now we have part-of, facet and similarity relations in the graph. After adding these links, the graph becomes highly connected. As shown in Fig. 12a, we reach a recall of 98% in the 6th step. The total graph has 363,252 nodes, of which we see 352,208 nodes. This way we reach approximately all nodes in the graph.

With this high recall value, again the questions arises whether we reach the same amount of recall by adding the same number of random links. We perform the same scenario of checking the contribution of added links for recall by comparison of adding the same number of random links. The number of 3,535,437 real similarity links is added in the graph between documents and between images. We add the same number of links randomly to the graph. We observe in Fig. 12a that we reach 99% of recall with random similarity links in the 5th step. After this step, there are few relevant images remaining to be reached. However, we should consider the expense of this high recall based on the number of visited nodes in each step. We compare the percentage of the graph seen in the other two lines in Fig. 12a for real and random similarity links. We observe a higher slope in the line with triangles, which reaches the 99% visit of the graph in the 5th step, whereas given real similarity links (the line with circles), we reach the same recall with visiting 98% of the graph. These percentages are near, but the question is that how much of the graph is needed to be visited to reach such recall values.

To answer, we show the expense of visiting this large number of nodes. We calculate the precision in each step in the graph with real/random similarity links. Figure 12b shows the precision loss in each step based on Eq. 4 as before. We observe that we lose more than 50% of precision in the first four steps. In steps 5, 6, 7 and 8, the recall value in both graphs is approximately equal. Therefore, the *precLoss* formula gives values near zero. In step 9 (the last step of visiting relevant nodes in the graph with random similarity links), there are not many relevant nodes remaining to be seen in the graph with random similarity links. This is because of the high connectivity in the graph with random similarity links. However, with real similarity links, there are still relevant nodes to be seen in this step, leading to larger value in the denominator of Eq. 4. We observe that although we visit a larger number of relevant nodes in pursuing random similarity links, the number of visited nodes is too high (e.g. 215,588 nodes in the 4th step). This leads to higher percentage of precision loss in the first steps. This analysis demonstrates the effective contribution of adding similarity links to the collection to increase the reachability of relevant nodes.

## Conclusion

We described the characteristics of our framework, Astera, which is a graph-based model for multimodal IR. Astera can model different types of data collections with various modalities and link types. It is able to enrich the modelled collection by extracting inherent features of information objects as facets; further by adding semantic/similarity links between information objects. We evaluated this model with the ImageCLEF2011 Wikipedia collection.

In the first part of the experiments, we presented a reachability analysis of relevant objects from different facets. We showed the effects that choosing diverse facets have on reachability. The results highlight that the combination of document and image textual facets results in better recall than the combination of document textual and image visual facets. Further, we highlight that using larger number of facets does not always lead to higher recall. We found that leveraging cognitively different facets (e.g. combination of document textual and image textual and visual facets) results in higher recall than functionally different ones (e.g. different document textual facets).

In addition, we designed experiments to investigate whether facets with the same recall behaviour visit different parts of the collection. We found that although the image visual facets show the same recall behaviour, they visit totally different relevant images at the initial steps 1–10. This encourages to leverage more than one facet to start traversing the graph from, as they help to see relevant nodes earlier. In addition, the analysis on textual facets showed that the language model (LM) facet leads to different parts of the graph than BM25 and TF.IDF facets (specially for image textual facets). The LM formula takes a probabilistic approach for computing the relevance, whereas TF.IDF and BM25 are both based on TF and IDF values. These results show that applying the poly-representation principle is important in identifying relevant information objects.

Further, we looked at the effect of different facets and links on different topic categories (easy, medium, hard, very hard). We observed that easy and medium topics are mainly reachable in initial steps, indicating that they are well served with standard similarity-based search. However, the relations in the graph helped significantly in reaching relevant images for hard and very hard topics. We observed a recall increase of 266% for hard and 373% for very hard topics. This observation emphasizes the role of the graph model to reach relevant information objects.

We conclude that adding a higher number of facets leads to much higher coverage of the graph and thus computational resource requirements. Therefore, the recall gain compared to the number of visited nodes is important. We observed that by using CEDD facet results, we visit a larger part of the graph with lower recall gain compared to results with TF.IDF facet of image textual annotations. Further, we observed that leveraging facets with different aspects of the information object lowers the number of steps to reach the recall plateau.

In the second part of our experiments, we showed the effect of adding semantic and similarity links in increasing the reachability in the graph. We gained 10% increase in recall by adding real semantic links, with the overhead of 22% increase in the number of nodes seen. This observation shows that we cannot easily increase recall, when the baseline is already 0.76. This highlights the importance of type and number of the links we choose to add. Further, we showed the contribution of adding similarity links to increase reachability to 98% of relevant information objects. In both cases, we compared the result in the graph with the same number of random links added. The rate of precision loss in the graph with random links showed the contribution of real semantic and similarity links to the graph.

While this paper presented solutions to a number of important issues, there are still some questions that could not be fully addressed. In the current version of Astera, we set the weights of different facets based on our analysis of their best combination. Further, we define weighting strategies for different relation types in our model. One future direction is to apply machine learning algorithms in both cases. Further is to create semantic facets for images in the collection. This way, concepts of each image are extracted by using concept detection methods [5]. These concepts are added as textual semantic facet to the images. Then, we can evaluate the effect of using semantic facets in reachability. Finally, it is worth looking into finding a set of facets of the query modalities that help contributing to a higher reachability and ideally each visit different parts of the graph.

## Footnotes

1
http://www.ifs.tuwien.ac.at/~sabetghadam/Astera.html.

2
https://Lucene.apache.org/.

3
http://www.lire-project.net/.

## References

[1] Atrey PK, Hossain MA, El Saddik A, Kankanhalli MS (2010). Multimodal fusion for multimedia analysis: a survey. J Multimed Syst.

[2] Berber T, Vahid AH, Ozturkmenoglu O, Hamed RG, Alpkocak A (2011) Demir at imageclefwiki 2011: evaluating different weighting schemes in information retrieval. In: Proceedings of conference and labs of the evaluation forum (CLEF)

[3] Berkhin P (2005). Survey: a survey on pagerank computing. J Internet Math.

[4] Bredin H, Roy A, Le V-B, Barras C (2014). Person instance graphs for mono-, cross-and multi-modal person recognition in multimedia data: application to speaker identification in TV broadcast. Int J Multimed Inf Retr (IJMIR).

[5] Budikova P, Botorek J, Batko M, Zezula P et al (2014) Disa at imageclef 2014: the search-based solution for scalable image annotation. In: CLEF (Working Notes), pp 360–371

[6] Carson C, Belongie S, Greenspan H, Malik J (2002). Blobworld: image segmentation using expectation-maximization and its application to image querying. IEEE Trans Pattern Anal Mach Intell.

[7] Chang, S-F, Ma W-Y, Smeulders, A (2007) Recent advances and challenges of semantic image/video search. In: Proceedings of the international conference on acoustics, speech and signal processing (ICASSP), vol 4, pp IV–1205

[8] Cheng P-C, Yeh J-Y, Ke H-R, Chien B-C, Yang W-P (2004) Comparison and combination of textual and visual features for interactive cross-language image retrieval. In: Workshop of the cross-language evaluation forum for european languages, Springer, pp 793–804

[9] Clements M, De Vries AP, Reinders MJ (2010). The task-dependent effect of tags and ratings on social media access. ACM Trans Inf Syst (TOIS).

[10] Collins-Thompson K, Callan J (2005) Query expansion using random walk models. In: Proceedings of Conference on Information and Knowledge Management (CIKM), pp 704–711

[11] Crestani F (1997). Application of spreading activation techniques in information retrieval. Artificial Intell Rev.

[12] Datta R, Joshi D, Li J, Wang JZ (2008). Image retrieval: ideas, influences, and trends of the new age. J ACM Comput Surv.

[13] Duan L, Li W, Tsang IW, Xu D (2011). Improving web image search by bag-based reranking. IEEE Trans Image Process.

[14] Elbassuoni S, Blanco R (2011) Keyword search over RDF graphs. In: Proceedings of conference on information and knowledge management (CIKM), pp 237–242

[15] Fergus R, Fei-Fei L, Perona P, Zisserman A (2005) Learning object categories from Google’s image search. In: Proceedings of international conference on computer vision, pp 1816–1823

[16] Fergus R, Perona P, Zisserman A (2004) A visual category filter for Google images. In: Proceedings of European conference on computer vision (ECCV), pp 242–256

[17] Hauptmann AG, Christel MG, Yan R (2008). Video retrieval based on semantic concepts. Proc IEEE.

[18] Hsu, WH, Kennedy, LS, Chang S-F (2007) Video search reranking through random walk over document-level context graph. In: Proceedings of the 15th ACM international conference on multimedia, pp 971–980

[19] Hwang SJ, Grauman K (2010) Accounting for the relative importance of objects in image retrieval. In: Proceedings of British machine vision conference (BMVC), pp 1–12

[20] Jing Y, Baluja S (2008). Visualrank: applying pagerank to large-scale image search. IEEE Trans Pattern Anal Mach Intell.

[21] Joshi D, Wang JZ, Li J (2006). The story picturing engine–a system for automatic text illustration. ACM Trans Multimed Comput Commun Appl (TOMM).

[22] Larsen B, Ingwersen P, Kekäläinen J (2006) The polyrepresentation continuum in IR. In: Proceedings of information interaction in context (IIiX), pp 88–96

[23] Lazaridis M, Axenopoulos A, Rafailidis D, Daras P (2013). Multimedia search and retrieval using multimodal annotation propagation and indexing techniques. J Signal Process Image Commun.

[24] Li X, Snoek CG, Worring M (2010) Unsupervised multi-feature tag relevance learning for social image retrieval. In: Proceedings of the ACM international conference on image and video retrieval, pp 10–17

[25] Liu J, Lai W, Hua X-S, Huang Y, Li S (2007) Video search re-ranking via multi-graph propagation. In: Proceedings of the 15th ACM international conference on multimedia, pp 208–217

[26] Ma W-Y, Manjunath BS (1999). Netra: a toolbox for navigating large image databases. J Multimed Syst.

[27] Martinet J, Satoh S (2007) An information theoretic approach for automatic document annotation from intermodal analysis. In: Proceedings of the workshop on multimodal information retrieval

[28] Mc Donald K, Smeaton AF (2005) A comparison of score, rank and probability-based fusion methods for video shot retrieval. In: Image and video retrieval, pp 61–70

[29] Mei T, Rui Y, Li S, Tian Q (2014). Multimedia search reranking: a literature survey. J ACM Comput Surv (CSUR).

[30] Minack E, Paiu R, Costache S, Demartini G, Gaugaz J, Ioannou E, Chirita P-A, Nejdl W (2010) Leveraging personal metadata for desktop search: the Beagle++ system. J Web Semant Sci Serv Agents WWW 8(1):37–54

[31] Nottelmann H, Fuhr N (2003) From retrieval status values to probabilities of relevance for advanced IR applications. J Inf Retr 6(3):363–388

[32] Ojala T, Pietikäinen M (1999). Unsupervised texture segmentation using feature distributions. J Pattern Recognit.

[33] Pentland A, Picard RW, Sclaroff S (1996). Photobook: content-based manipulation of image databases. Int J Comput Vis.

[34] Rocha C, Schwabe D, Aragao MP (2004) A hybrid approach for searching in the semantic web. In: Proceedings of world wide web conference (WWW), pp 374–383

[35] Sabetghadam S, Bierig R, Rauber A (2014) A hybrid approach for multi-faceted IR in multimodal domain. In: Proceedings of conference and labs of the evaluation forum (CLEF), pp 86–97

[36] Sabetghadam S, Lupu M, Bierig R, Rauber A (2014) A combined approach of structured and non-structured IR in multimodal domain. In: Proceedings of international conference on multimedia retrieval (ICMR), pp 491–495

[37] Sabetghadam S, Lupu M, Bierig R, Rauber A (2015) Reachability analysis of graph modelled collections. In: European conference on information retrieval (ECIR), pp 370–381 10.1007/s13735-017-0145-8 PMC6417456 30956928

[38] Sabetghadam S, Lupu M, Rauber A (2013) Astera—a generic model for multimodal information retrieval. In: Proceedings of integrating IR technologies for professional search workshop

[39] Sabetghadam S, Lupu M, Rauber A (2014) Which one to choose: random walk or spreading activation? In: Proceedings of information retrieval facility conference (IRFC), pp 112–119

[40] Schinas M, Papadopoulos S, Kompatsiaris Y, Mitkas PA (2016). Mgraph: multimodal event summarization in social media using topic models and graph-based ranking. Int J Multimed Inf Retr (IJMIR).

[41] Skov M, Larsen B, Ingwersen P (2008). Inter and intra-document contexts applied in polyrepresentation for best match IR. J Inf Process Manag.

[42] Smeulders AW, Worring M, Santini S, Gupta A, Jain R (2000). Content-based image retrieval at the end of the early years. IEEE Trans Pattern Anal Mach Intell.

[43] Stehling RO, Nascimento MA, Falcão AX (2002) A compact and efficient image retrieval approach based on border/interior pixel classification. In: Proceedings of conference on information and knowledge management (CIKM), pp 102–109

[44] Szummer M and Jaakkola T (2001) Partially labeled classification with markov random walks. In: Proceedings of neural information processing systems (NIPS), pp 945–952

[45] Thomee B, Lew MS (2012). Interactive search in image retrieval: a survey. Int J Multimed Inf Retr (IJMIR).

[46] Tonon A, Demartini G, Cudré-Mauroux P (2012) Combining inverted indices and structured search for ad-hoc object retrieval. In: Proceedings of special interest group on information retrieval (SIGIR), pp 125–134

[47] Tsikrika T, Popescu A, Kludas J (2011) Overview of the Wikipedia image retrieval task at imageclef 2011. In: Conference and labs of the evaluation forum (CLEF)

[48] Wang M, Li H, Tao D, Lu K, Wu X (2012). Multimodal graph-based reranking for web image search. IEEE Trans Image Process.

[49] Yao T, Mei T, Ngo C-W (2010) Co-reranking by mutual reinforcement for image search. In: Proceedings of the ACM international conference on image and video retrieval, pp 34–41

